# Supplementary material for: The Associations of Maternal Hemoglobin Concentration in Different Time Points and Its Changes during Pregnancy with Birth Weight Outcomes
Source: Nutrients. 2022 Jun 19;14(12):2542. doi: 10.3390/nu14122542 (PMC9229552; doi:10.3390/nu14122542)
Supplement: Supplementary file 1 [file nutrients-14-02542-s001.zip › supplementary Figures.pdf]

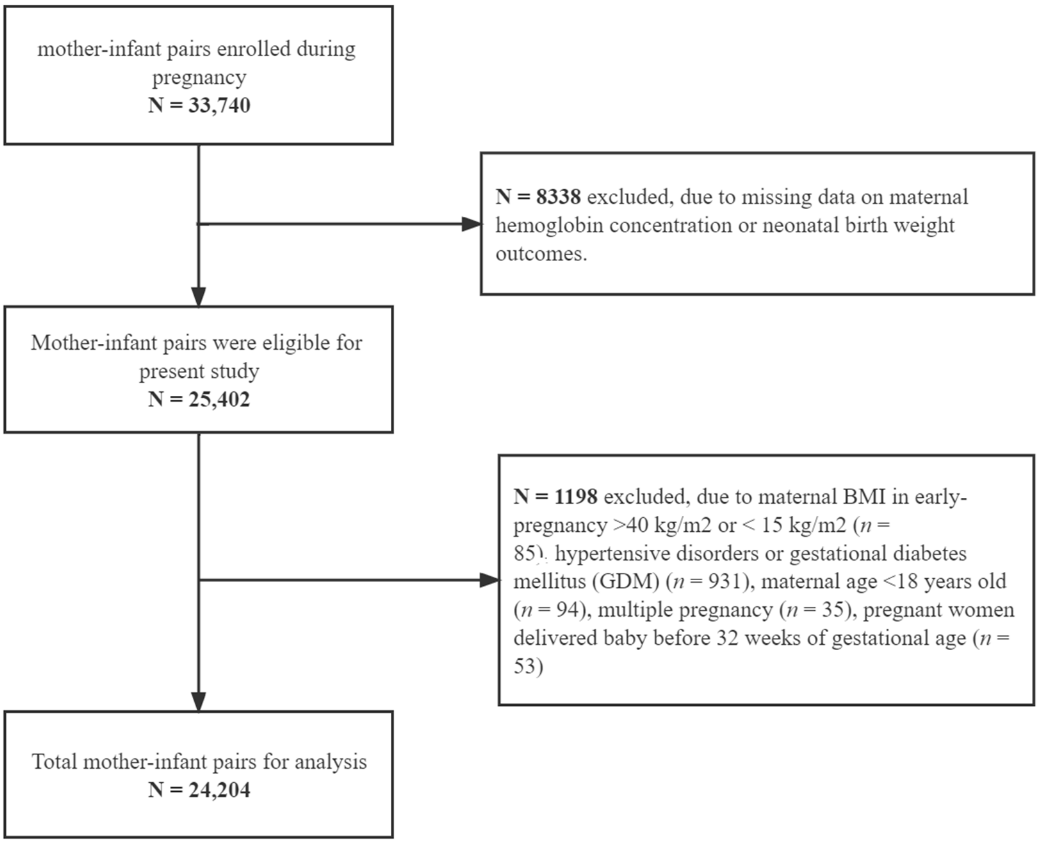

Figure S1. The flow chart of participants.

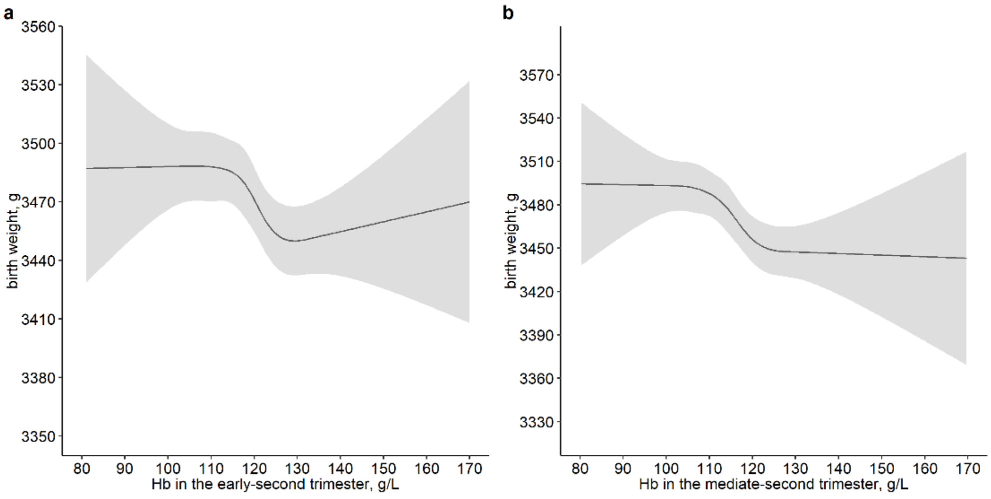

Figure S2. Dose–response association of maternal hemoglobin in the early-second trimester (**a**) and the mediate-second trimester (**b**) with neonatal birth weight. The *p*-values for overall associations were both >0.05. The *p*-values for non-linear associations were 0.062 (**a**) and 0.053 (**b**). Models were adjusted for maternal age, education, parity, gestational age of hemoglobin measurement, neonatal.

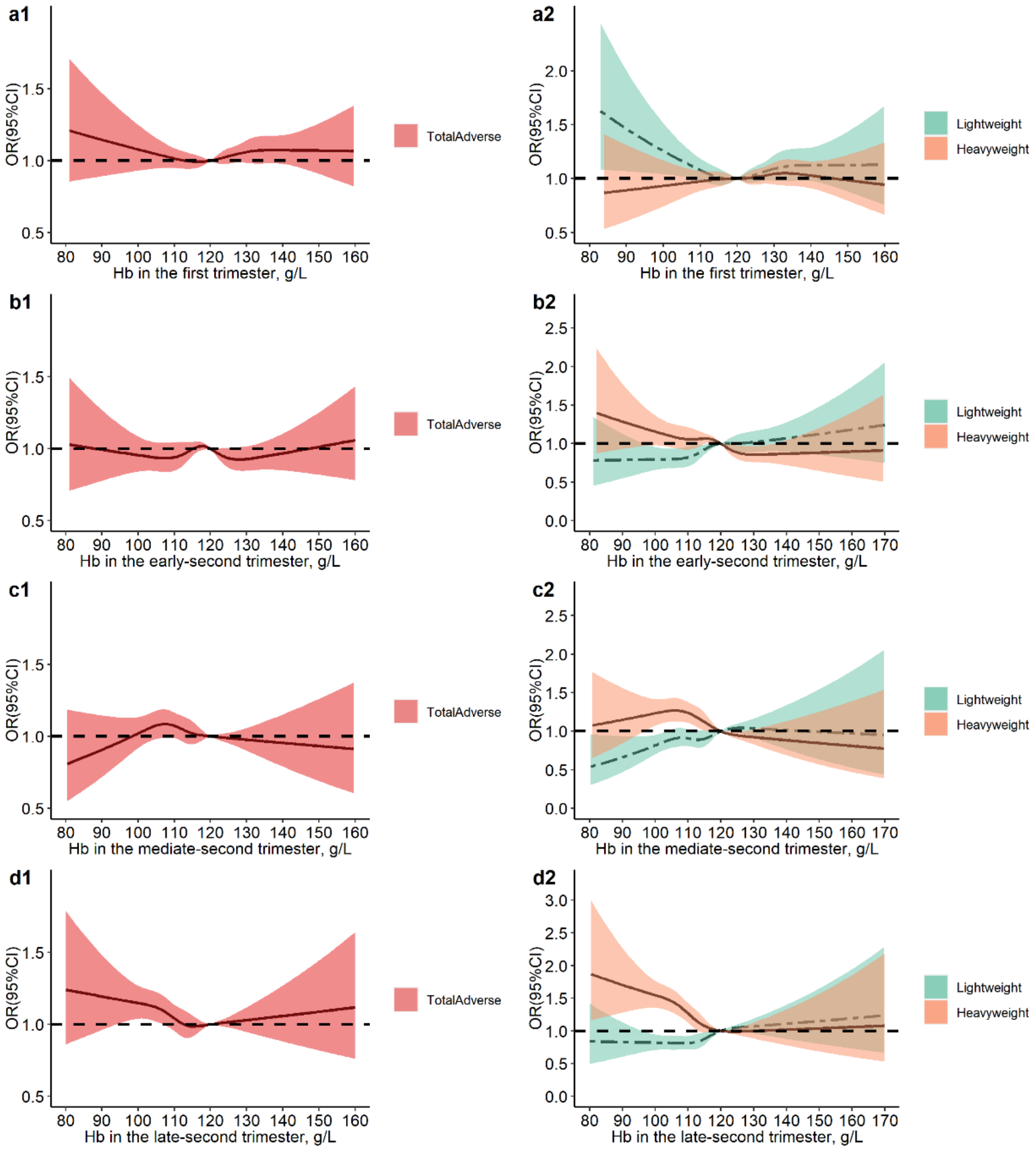

Figure S3. Dose–response association of maternal hemoglobin in the first trimester (**a**), early-second trimester (**b**), mediate-second trimester (**c**) and late-second trimester (**d**) with neonatal complex outcomes (including SGA/LBW, LGA/macrosomia and total birth weight adverse outcomes). The *p*-values for non-linear associations between Hb and total adverse outcomes were 0.459 (**a1**), 0.316 (**b1**), 0.340 (**c1**) and 0.146 (**d1**); the *p*-values for non-linear associations between Hb and SGA/LBW were 0.070 (**a2**), 0.494 (**b2**), 0.469 (**c2**) and 0.251 (**d2**); and the ***P***-values for non-linear associations between Hb and LGA/macrosomia were 0.869 (**a2**), 0.360 (**b2**), 0.160 (**c2**) and 0.085 (**d2**). Models were adjusted for maternal age, education, parity, gestational age of hemoglobin measurement, neonatal gender, gestational age at delivery, mode of delivery, maternal BMI in the first trimester and weight gain during pregnancy. Hemoglobin at 120 g/L was set as the reference level.

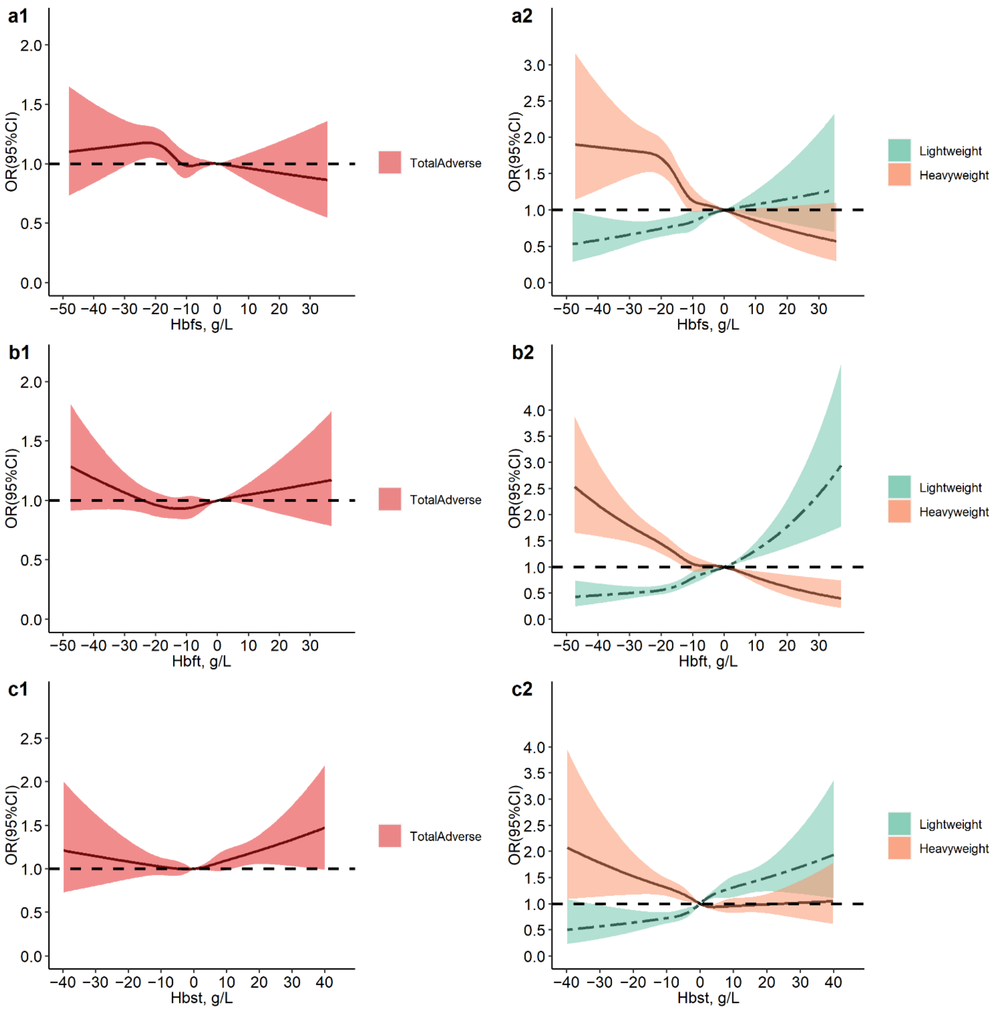

Figure S4. Dose–response association of maternal hemoglobin change from the first to late-second trimesters (**a**), first to late-third trimesters (**b**) and late-second to late-third trimesters (**c**) with neonatal complex outcomes (including SGA/LBW, LGA/macrosomia and total birth weight adverse outcomes). The *p*-values for non-linear associations between Hb change and total adverse outcomes were 0.184 (**a1**), 0.051 (**b1**) and 0.216 (**c1**); the *p*-values for non-linear associations between Hb change and SGA/LBW were 0.863 (**a2**), 0.345 (**b2**) and 0.113 (**c2**); and the *p*-values for non-linear associations between Hb change and LGA/macrosomia were 0.226 (**a2**), 0.178 (**b2**) and 0.159 (**c2**). Models were adjusted for maternal age, education, parity, neonatal gender, gestational age at delivery, mode of delivery, maternal BMI in the first trimester, weight gain during pregnancy, and Hb concentration in the first trimester or late-second trimester. A hemoglobin change of 0 g/L was set as the reference level. Hbfs, Hb in late-second minus Hb in first; Hbft, Hb in late-third minus Hb in first; Hbst, Hb in late-third minus Hb in late-second.
