# Supplementary material for: The Associations of Maternal Hemoglobin Concentration in Different Time Points and Its Changes during Pregnancy with Birth Weight Outcomes
Source: Nutrients. 2022 Jun 19;14(12):2542. doi: 10.3390/nu14122542 (PMC9229552; doi:10.3390/nu14122542)
Supplement: Supplementary file 1 [file nutrients-14-02542-s001.zip › supplementary Tables.pdf]

Table S1. The comparison of general information between participants and non-participants.

Variables	Non-Participants N = 10,456	Participants N = 24,183	*p*
Maternal age, years, mean ± SD	28.51 (3.98)	26.64 (3.72)	<0.001
Education, N (%)			
Primary school or below	149 (1.4)	441 (1.8)	<0.001
Junior high school	1793 (17.3)	8270 (34.5)	
Senior high school	2115 (20.4)	6074 (25.4)	
College or above	6333 (61.0)	9174 (38.3)	
Primipara, N (%)			
Yes	6857 (65.6)	14,481 (59.9)	<0.001
No	2984 (28.5)	2360 (9.8)	
Unknown	615 (5.9)	7342 (30.4)	
BMI at 1st trimester, kg/m^2, mean ± SD	21.38 (3.09)	20.67 (2.61)	<0.001
Gender of newborn, N (%)			
Male	5406 (51.7)	12,855 (53.2)	0.013
Female	5050 (48.3)	11,328 (46.8)	
Mode of delivery, N (%)			
Vaginal	5333 (51.0)	9393 (38.8)	<0.001
Cesarean	4542 (43.4)	13,463 (55.7)	
Forceps	184 (1.8)	260 (1.1)	
Others	397 (3.8)	1067 (4.4)	
Preterm, N (%)			
No	9966 (95.3)	23,445 (96.9)	<0.001
Yes	490 (4.7)	738 (3.1)	

Table S2. Maternal hemoglobin status in different periods of pregnancy and the change during pregnancy between groups.

Hemoglobin	Normalweight N = 19,425	Lightweight N = 2063	Heavyweight N = 2716	*p*
**Maternal Hb Status**				
First trimester (*n* = 24,204)				
Average, g/L, mean ± SD	123.88 ± 10.09	123.61 ± 10.76	124.78 ± 9.92	<0.001
Hb status, N (%)				
<100	189 (1.0)	30 (1.5)	17 (0.6)	0.001
100~109	1136 (5.9)	136 (6.6)	139 (5.1)	
110~119	4830 (24.9)	501 (24.4)	613 (22.6)	
120~129	7911 (40.8)	821 (40.0)	1115 (41.1)	
130~139	4356 (22.4)	447 (21.8)	665 (24.5)	
≥140	991 (5.1)	120 (5.8)	166 (6.1)	
Early-second trimester (*n* = 19,828)				
Average, g/L, mean ± SD	117.90 ± 9.84	118.31 ± 10.14	118.09 ± 9.61	0.203
Hb status, N (%)				
<100	381 (2.4)	43 (2.6)	60 (2.7)	0.694
100~109	2445 (15.3)	230 (13.9)	328 (14.7)	
110~119	6407 (40.2)	672 (40.5)	869 (39.0)	

120~129	5119 (32.1)	533 (32.1)	752 (33.7)	
130~139	1323 (8.3)	147 (8.9)	182 (8.2)	
≥140	266 (1.7)	33 (2.0)	38 (1.7)	
Mediate-second trimester (*n* = 21,495)				
Average, g/L, mean ± SD	114.29 ± 9.09	114.43 ± 8.88	114.39 ± 8.67	0.728
Hb status, N (%)				
<100	694 (4.0)	73 (4.0)	83 (3.4)	0.731
100~109	4268 (24.8)	450 (24.5)	608 (25.1)	
110~119	7865 (45.6)	815 (44.3)	1130 (46.7)	
120~129	3720 (21.6)	428 (23.3)	500 (20.7)	
130~139	588 (3.4)	61 (3.3)	81 (3.3)	
≥140	104 (0.6)	11 (0.6)	16 (0.7)	
Late-second trimester (*n* = 21,432)				
Average, g/L, mean ± SD	113.27 ± 8.84	113.58 ± 8.93	112.92 ± 8.61	0.048
Hb status, N (%)				
<100	714 (4.1)	83 (4.6)	111 (4.6)	0.028
100~109	4885 (28.4)	497 (27.3)	733 (30.5)	
110~119	8036 (46.7)	820 (45.0)	1068 (44.5)	
120~129	3097 (18.0)	351 (19.3)	412 (17.2)	
130~139	395 (2.3)	60 (3.3)	67 (2.8)	
≥140	83 (0.5)	10 (0.5)	10 (0.4)	
Early-third trimester (*n* = 21,093)				
Average, g/L, mean ± SD	113.24 ± 8.63	114.57 ± 9.12	112.70 ± 8.63	<0.001
Hb status, N (%)				
<100	716 (4.2)	58 (3.2)	104 (4.4)	<0.001
100~109	4929 (29.2)	442 (24.5)	794 (33.3)	
110~119	7698 (45.5)	806 (44.6)	1012 (42.4)	
120~129	3007 (17.8)	429 (23.8)	396 (16.6)	
130~139	458 (2.7)	60 (3.3)	61 (2.6)	
≥140	94 (0.6)	11 (0.6)	18 (0.8)	
Late-third trimester (*n* = 23,140)				
Average, g/L, mean ± SD	114.95 ± 8.70	116.55 ± 9.21	114.27 ± 9.53	<0.001
Hb status, N (%)				
<100	529 (2.8)	49 (2.6)	91 (3.5)	<0.001
100~109	4541 (24.3)	375 (19.8)	741 (28.5)	
110~119	8534 (45.8)	819 (43.3)	1121 (43.2)	
120~129	4080 (21.9)	496 (26.2)	503 (19.4)	
130~139	849 (4.6)	131 (6.9)	119 (4.6)	
≥140	119 (0.6)	22 (1.2)	21 (0.8)	
**Change in Maternal Hb Concentration**				
Hbfs (*n* = 21,389)				
Average, g/L, mean ± SD	−10.67 ± 9.47	−10.20 ± 9.53	−11.84 ± 9.43	<0.001
Categories, N (%)				
Q1 (−50~−17)	4332 (25.2)	444 (24.4)	724 (30.2)	<0.001
Q2 (−17~−11)	4402 (25.6)	429 (23.6)	642 (26.8)	

Q3 (−11~−5)	4407 (25.7)	476 (26.2)	537 (22.4)	
Q4 (−5~50)	4034 (23.5)	467 (25.7)	495 (20.6)	
Hbft (*n* = 23,103)				
Average, g/L, mean ± SD	−8.89 ± 11.00	−6.96 ± 11.43	−10.51 ± 11.11	<0.001
Categories, N (%)				
Q1 (−50~−16)	4906 (26.3)	391 (20.7)	831 (32.1)	<0.001
Q2 (−16~−9)	4572 (24.6)	438 (23.2)	626 (24.2)	
Q3 (−9~−2)	4527 (24.3)	477 (25.2)	579 (22.3)	
Q4 (−2~50)	4617 (24.8)	584 (30.9)	555 (21.4)	
Hbst (*n* = 20,543)				
Average, g/L, mean ± SD	1.77 ± 8.46	3.10 ± 8.52	1.35 ± 8.69	<0.001
Categories, N (%)				
Q1 (−50~−3.5)	4362 (26.3)	347 (20.7)	678 (29.5)	<0.001
Q2 (−3.5~2)	4477 (27.0)	431 (25.7)	610 (26.6)	
Q3 (2~7)	3727 (22.5)	405 (24.1)	454 (19.8)	
Q4 (7~50)	4000 (24.1)	497 (29.6)	555 (24.2)	

Lightweight, small for gestational age (SGA) or low birth weight (LBW); Heavyweight, large for gestational age (LGA) or macrosomia; Hbfs, Hb in late-second minus Hb in first; Hbft, Hb in late-third minus Hb in late-third; Hbst, Hb in late-second minus Hb in late-third; Q, quartile.

Table S3. Associations between maternal hemoglobin concentrations in different periods and adverse birth outcomes in women with early-pregnancy BMI < 24 kg/m².

Hemoglobin	Normalweight	Lightweight			Heavyweight		
	N (%)	N (%)	Model 1 † OR (95% CI)	Model 2 ‡ OR (95% CI)	N (%)	Model 1 † OR (95% CI)	Model 2 ‡ OR (95% CI)
First trimester							
<100	178 (1.0)	27 (1.4)	1.41 (0.92, 2.15)	1.36 (0.89, 2.08)	16 (0.7)	0.82 (0.48, 1.38)	0.83 (0.49, 1.42)
100~109	1085 (6.1)	132 (6.9)	1.16 (0.94, 1.42)	1.13 (0.92, 1.40)	124 (5.7)	1.01 (0.82, 1.25)	1.07 (0.86, 1.32)
110~119	4599 (26.0)	478 (25.0)	Ref.	Ref.	531 (24.5)	Ref.	Ref.
120~129	7211 (40.7)	768 (40.2)	1.01 (0.90, 1.14)	1.05 (0.92, 1.18)	898 (41.4)	1.08 (0.97, 1.22)	1.04 (0.93, 1.17)
130~139	3816 (21.6)	404 (21.1)	1.03 (0.89, 1.18)	1.07 (0.93, 1.24)	497 (22.9)	1.12 (0.98, 1.28)	1.07 (0.94, 1.23)
≥140	809 (4.6)	102 (5.3)	1.21 (0.96, 1.52)	1.25 (0.99, 1.59)	104 (4.8)	1.08 (0.86, 1.36)	0.98 (0.77, 1.24)
Early-second trimester							
<100	363 (2.5)	40 (2.6)	0.97 (0.69, 1.37)	0.91 (0.64, 1.29)	53 (3.0)	1.24 (0.91, 1.69)	1.34 (0.98, 1.83)
100~109	2356 (16.2)	222 (14.4)	0.88 (0.75, 1.03)	0.85 (0.72, 1.00)	288 (16.2)	1.01 (0.87, 1.17)	1.07 (0.92, 1.25)
110~119	5960 (41.0)	640 (41.5)	Ref.	Ref.	728 (41.0)	Ref.	Ref.
120~129	4527 (31.2)	485 (31.4)	1.00 (0.88, 1.14)	1.05 (0.92, 1.19)	564 (31.8)	1.00 (0.88, 1.12)	0.94 (0.83, 1.06)
130~139	1104 (7.6)	127 (8.2)	1.09 (0.89, 1.34)	1.17 (0.95, 1.45)	119 (6.7)	0.85 (0.69, 1.04)	0.76 (0.62, 0.95)
≥140	218 (1.5)	29 (1.9)	1.18 (0.78, 1.76)	1.26 (0.83, 1.90)	22 (1.2)	0.88 (0.56, 1.38)	0.79 (0.50, 1.26)
Mediate-second trimester							
<100	667 (4.2)	71 (4.1)	0.99 (0.76, 1.28)	0.89 (0.68, 1.15)	74 (3.8)	0.91 (0.70, 1.17)	1.03 (0.79, 1.34)
100~109	4039 (25.7)	428 (25.0)	1.01 (0.89, 1.14)	0.96 (0.84, 1.09)	533 (27.5)	1.05 (0.94, 1.18)	1.15 (1.03, 1.30)
110~119	7207 (45.8)	759 (44.3)	Ref.	Ref.	911 (47.0)	Ref.	Ref.
120~129	3219 (20.5)	387 (22.6)	1.13 (0.99, 1.29)	1.19 (1.04, 1.36)	363 (18.7)	0.89 (0.78, 1.02)	0.84 (0.73, 0.96)
130~139	492 (3.1)	57 (3.3)	1.15 (0.86, 1.53)	1.19 (0.89, 1.60)	44 (2.3)	0.69 (0.50, 0.96)	0.65 (0.47, 0.90)
≥140	95 (0.6)	10 (0.6)	0.94 (0.48, 1.84)	1.02 (0.52, 2.02)	13 (0.7)	1.10 (0.61, 2.00)	0.95 (0.52, 1.75)
Late-second trimester							
<100	666 (4.2)	79 (4.7)	1.11 (0.86, 1.42)	0.99 (0.77, 1.27)	95 (5.0)	1.29 (1.02, 1.62)	1.52 (1.20, 1.92)
100~109	4629 (29.5)	472 (27.8)	0.97 (0.86, 1.09)	0.91 (0.81, 1.03)	641 (33.5)	1.21 (1.08, 1.35)	1.32 (1.17, 1.47)
110~119	7322 (46.7)	764 (45.1)	Ref.	Ref.	851 (44.5)	Ref.	Ref.
120~129	2669 (17.0)	318 (18.8)	1.15 (1.00, 1.33)	1.23 (1.06, 1.41)	281 (14.7)	0.90 (0.78, 1.04)	0.84 (0.73, 0.98)
130~139	338 (2.2)	53 (3.1)	1.49 (1.10, 2.03)	1.56 (1.14, 2.14)	36 (1.9)	0.96 (0.67, 1.37)	0.89 (0.62, 1.28)
≥140	70 (0.4)	9 (0.5)	1.09 (0.53, 2.23)	1.23 (0.59, 2.55)	7 (0.4)	0.96 (0.44, 2.11)	0.83 (0.37, 1.85)

Early-third trimester							
<100	674 (4.4)	54 (3.2)	0.72 (0.54, 0.96)	0.65 (0.49, 0.87)	91 (4.8)	1.18 (0.93, 1.49)	1.32 (1.04, 1.69)
100~109	4620 (30.0)	422 (25.1)	0.83 (0.73, 0.94)	0.80 (0.70, 0.90)	674 (35.3)	1.26 (1.13, 1.41)	1.35 (1.20, 1.51)
110~119	7022 (45.6)	754 (44.9)	Ref.	Ref.	815 (42.6)	Ref.	Ref.
120~129	2618 (17.0)	385 (22.9)	1.34 (1.17, 1.53)	1.39 (1.22, 1.60)	279 (14.6)	0.92 (0.79, 1.06)	0.85 (0.73, 0.98)
130~139	394 (2.6)	54 (3.2)	1.19 (0.88, 1.61)	1.27 (0.94, 1.73)	43 (2.3)	0.92 (0.66, 1.28)	0.86 (0.61, 1.20)
≥140	86 (0.6)	10 (0.6)	1.02 (0.52, 2.01)	1.01 (0.51, 2.00)	9 (0.5)	0.93 (0.46, 1.86)	0.87 (0.42, 1.79)
Late-third trimester							
<100	495 (2.9)	46 (2.6)	0.95 (0.69, 1.30)	0.90 (0.65, 1.23)	79 (3.8)	1.33 (1.03, 1.71)	1.43 (1.10, 1.85)
100~109	4266 (25.1)	355 (20.0)	0.84 (0.74, 0.96)	0.82 (0.71, 0.93)	619 (29.7)	1.23 (1.10, 1.38)	1.27 (1.13, 1.42)
110~119	7802 (45.8)	773 (43.6)	Ref.	Ref.	910 (43.7)	Ref.	Ref.
120~129	3633 (21.3)	455 (25.7)	1.26 (1.11, 1.42)	1.29 (1.14, 1.47)	382 (18.4)	0.92 (0.81, 1.04)	0.86 (0.75, 0.98)
130~139	723 (4.2)	123 (6.9)	1.69 (1.37, 2.09)	1.88 (1.52, 2.32)	78 (3.7)	0.92 (0.72, 1.18)	0.83 (0.64, 1.06)
≥140	99 (0.6)	19 (1.1)	1.96 (1.19, 3.25)	2.04 (1.22, 3.40)	13 (0.6)	1.14 (0.63, 2.06)	0.98 (0.53, 1.80)

Lightweight, small for gestational age (SGA) or low birth weight (LBW); Heavyweight, large for gestational age (LGA) or macrosomia. † The model was adjusted for maternal age, education, parity, gestational age of hemoglobin measurement, neonatal gender, gestational age at delivery and mode of delivery. ‡ The model was additionally adjusted for maternal BMI in the first trimester and weight gain during pregnancy.

Table S4. Associations between change in maternal hemoglobin concentrations and adverse birth outcomes in women with early-pregnancy BMI < 24 kg/m^2.

ΔHb	Normalweight	Lightweight			Heavyweight		
	N (%)	N (%)	Model 1 † OR (95% CI)	Model 2 ‡ OR (95% CI)	N (%)	Model 1 † OR (95% CI)	Model 2 ‡ OR (95% CI)
Hbfs							
Q1 (−50~−17)	3893 (24.9)	397 (23.5)	Ref.	Ref.	563 (29.5)	Ref.	Ref.
Q2 (−17~−11)	3989 (25.5)	406 (24.0)	1.00 (0.86, 1.16)	1.04 (0.89, 1.21)	516 (27.0)	0.90 (0.78, 1.03)	0.83 (0.73, 0.96)
Q3 (−11~−5)	4029 (25.7)	447 (26.4)	1.12 (0.96, 1.31)	1.22 (1.04, 1.43)	421 (22.1)	0.71 (0.61, 0.82)	0.63 (0.54, 0.74)
Q4 (−5~50)	3753 (24.0)	440 (26.0)	1.22 (1.02, 1.45)	1.38 (1.15, 1.65)	408 (21.4)	0.71 (0.60, 0.84)	0.59 (0.50, 0.71)
Hbft							
Q1 (−50~−16)	4397 (25.9)	362 (20.5)	Ref.	Ref.	653 (31.5)	Ref.	Ref.
Q2 (−16~−9)	4144 (24.4)	405 (22.9)	1.30 (1.11, 1.51)	1.32 (1.13, 1.54)	499 (24.0)	0.80 (0.70, 0.91)	0.78 (0.68, 0.89)
Q3 (−9~−2)	4153 (24.4)	449 (25.4)	1.48 (1.26, 1.74)	1.56 (1.33, 1.83)	464 (22.4)	0.73 (0.64, 0.85)	0.69 (0.60, 0.80)
Q4 (−2~50)	4296 (25.3)	553 (31.3)	1.87 (1.57, 2.22)	2.01 (1.68, 2.39)	460 (22.2)	0.70 (0.60, 0.83)	0.63 (0.54, 0.75)
Hbst *							
Q1 (−50~−3.5)	3959 (26.2)	320 (20.3)	Ref.	Ref.	521 (28.4)	Ref.	Ref.
Q2 (−3.5~2)	4062 (26.9)	410 (26.0)	1.22 (1.04, 1.42)	1.21 (1.04, 1.42)	483 (26.3)	0.93 (0.82, 1.07)	0.96 (0.84, 1.10)
Q3 (2~7)	3410 (22.6)	376 (23.9)	1.34 (1.15, 1.58)	1.33 (1.14, 1.57)	364 (19.8)	0.83 (0.71, 0.95)	0.84 (0.73, 0.98)
Q4 (7~50)	3684 (24.4)	469 (29.8)	1.52 (1.31, 1.77)	1.47 (1.26, 1.72)	467 (25.4)	1.01 (0.88, 1.16)	1.07 (0.93, 1.23)

Lightweight, small for gestational age (SGA) or low birth weight (LBW); Heavyweight, large for gestational age (LGA) or macrosomia; Hbfs, Hb in late-second minus Hb in first; Hbft, Hb in late-third minus Hb in late-third; Hbst, Hb in late-second minus Hb in late-third; Q, quartile. † The model was adjusted for maternal age, education, parity, weeks of gestation at hemoglobin measurement, neonatal gender, gestational age at delivery, mode of delivery and Hb concentration in the first trimester. ‡ The model was additionally adjusted for maternal BMI in the first trimester and weight gain during pregnancy. * Model 1 was adjusted for maternal age, education, parity, weeks of gestation at hemoglobin measurement, neonatal gender, gestational age at delivery, mode of delivery and Hb concentration in the late-second trimester.

Table S5. Associations between maternal hemoglobin concentration in different periods and adverse birth outcomes in pregnant women who delivered their baby after 37 weeks of gestational age.

Hemoglobin	Normalweight	Lightweight			Heavyweight		
	N (%)	N (%)	Model 1 † OR (95% CI)	Model 2 ‡ OR (95% CI)	N (%)	Model 1 † OR (95% CI)	Model 2 ‡ OR (95% CI)
First trimester							
<100	188 (1.0)	23 (1.3)	1.30 (0.83, 2.03)	1.25 (0.79, 1.96)	16 (0.6)	0.69 (0.41, 1.17)	0.69 (0.41, 1.18)
100~109	1111 (5.8)	123 (7.0)	1.19 (0.96, 1.48)	1.17 (0.94, 1.44)	134 (5.1)	0.96 (0.79, 1.18)	1.02 (0.83, 1.26)
110~119	4742 (24.9)	439 (24.9)	Ref.	Ref.	595 (22.6)	Ref.	Ref.
120~129	7749 (40.7)	707 (40.2)	0.99 (0.87, 1.12)	1.03 (0.91, 1.17)	1087 (41.2)	1.11 (1.00, 1.24)	1.03 (0.92, 1.15)
130~139	4286 (22.5)	368 (20.9)	0.94 (0.82, 1.09)	1.01 (0.87, 1.17)	643 (24.4)	1.16 (1.03, 1.31)	1.04 (0.92, 1.18)
≥140	972 (5.1)	100 (5.7)	1.13 (0.90, 1.43)	1.26 (1.00, 1.60)	162 (6.1)	1.28 (1.05, 1.55)	1.04 (0.86, 1.28)
Early-second trimester							
<100	371 (2.4)	37 (2.6)	1.05 (0.74, 1.49)	0.97 (0.68, 1.38)	58 (2.7)	1.21 (0.90, 1.63)	1.34 (0.99, 1.82)
100~109	2401 (15.3)	203 (14.3)	0.91 (0.77, 1.08)	0.87 (0.73, 1.03)	319 (14.8)	1.02 (0.88, 1.17)	1.13 (0.98, 1.30)
110~119	6289 (40.2)	580 (40.9)	Ref.	Ref.	845 (39.1)	Ref.	Ref.
120~129	5031 (32.2)	446 (31.5)	0.97 (0.85, 1.10)	1.03 (0.90, 1.18)	723 (33.5)	1.04 (0.94, 1.16)	0.94 (0.84, 1.06)
130~139	1297 (8.3)	127 (9.0)	1.09 (0.89, 1.33)	1.23 (1.00, 1.52)	177 (8.2)	0.97 (0.81, 1.16)	0.80 (0.66, 0.96)
≥140	258 (1.6)	25 (1.8)	1.06 (0.70, 1.62)	1.21 (0.79, 1.86)	37 (1.7)	1.07 (0.75, 1.54)	0.88 (0.60, 1.28)
Mediate-second trimester							
<100	675 (4.0)	66 (4.2)	1.03 (0.79, 1.34)	0.91 (0.69, 1.19)	81 (3.5)	0.88 (0.69, 1.13)	1.05 (0.82, 1.35)
100~109	4197 (24.8)	384 (24.4)	0.99 (0.87, 1.13)	0.93 (0.81, 1.06)	593 (25.3)	1.01 (0.91, 1.13)	1.13 (1.01, 1.27)
110~119	7727 (45.7)	706 (44.8)	Ref.	Ref.	1090 (46.5)	Ref.	Ref.
120~129	3645 (21.5)	361 (22.9)	1.09 (0.95, 1.24)	1.16 (1.01, 1.33)	484 (20.7)	0.93 (0.82, 1.04)	0.83 (0.73, 0.93)
130~139	580 (3.4)	50 (3.2)	1.00 (0.74, 1.35)	1.09 (0.80, 1.48)	79 (3.4)	0.93 (0.73, 1.19)	0.79 (0.61, 1.03)
≥140	100 (0.6)	8 (0.5)	0.87 (0.42, 1.81)	0.97 (0.47, 2.03)	16 (0.7)	1.15 (0.67, 1.98)	0.99 (0.57, 1.73)
Late-second trimester							
<100	703 (4.2)	72 (4.6)	1.14 (0.88, 1.47)	1.00 (0.77, 1.30)	107 (4.6)	1.24 (1.00, 1.54)	1.49 (1.19, 1.86)
100~109	4787 (28.3)	432 (27.8)	1.00 (0.88, 1.13)	0.93 (0.81, 1.05)	717 (30.8)	1.16 (1.05, 1.29)	1.31 (1.17, 1.46)
110~119	7902 (46.8)	697 (44.8)	Ref.	Ref.	1030 (44.2)	Ref.	Ref.
120~129	3039 (18.0)	297 (19.1)	1.13 (0.98, 1.30)	1.23 (1.06, 1.42)	401 (17.2)	0.99 (0.87, 1.12)	0.87 (0.77, 1.00)
130~139	388 (2.3)	49 (3.2)	1.48 (1.08, 2.02)	1.62 (1.18, 2.23)	65 (2.8)	1.32 (1.00, 1.74)	1.09 (0.82, 1.45)
≥140	80 (0.5)	8 (0.5)	1.07 (0.51, 2.24)	1.21 (0.58, 2.54)	10 (0.4)	1.07 (0.54, 2.08)	0.95 (0.48, 1.89)

Early-third trimester							
<100	703 (4.2)	44 (2.9)	0.65 (0.47, 0.90)	0.58 (0.42, 0.80)	100 (4.3)	1.11 (0.89, 1.40)	1.30 (1.03, 1.64)
100~109	4824 (29.1)	380 (24.8)	0.83 (0.73, 0.95)	0.78 (0.68, 0.89)	777 (33.5)	1.25 (1.13, 1.39)	1.36 (1.22, 1.51)
110~119	7585 (45.7)	702 (45.7)	Ref.	Ref.	977 (42.2)	Ref.	Ref.
120~129	2935 (17.7)	353 (23.0)	1.32 (1.16, 1.52)	1.41 (1.23, 1.62)	385 (16.6)	1.00 (0.88, 1.14)	0.88 (0.77, 1.00)
130~139	442 (2.7)	46 (3.0)	1.18 (0.86, 1.62)	1.27 (0.92, 1.75)	61 (2.6)	1.03 (0.78, 1.36)	0.92 (0.69, 1.23)
≥140	93 (0.6)	10 (0.7)	1.15 (0.60, 2.23)	1.14 (0.58, 2.22)	16 (0.7)	1.40 (0.81, 2.41)	1.20 (0.67, 2.14)
Late-third trimester							
<100	522 (2.8)	35 (2.1)	0.78 (0.55, 1.10)	0.72 (0.51, 1.03)	91 (3.6)	1.26 (0.99, 1.60)	1.41 (1.10, 1.80)
100~109	4460 (24.3)	343 (20.3)	0.86 (0.75, 0.98)	0.82 (0.72, 0.94)	729 (28.8)	1.23 (1.11, 1.36)	1.29 (1.16, 1.44)
110~119	8415 (45.8)	743 (44.0)	Ref.	Ref.	1097 (43.3)	Ref.	Ref.
120~129	4013 (21.9)	441 (26.1)	1.29 (1.13, 1.46)	1.34 (1.18, 1.53)	481 (19.0)	0.92 (0.82, 1.03)	0.83 (0.74, 0.94)
130~139	830 (4.5)	110 (6.5)	1.55 (1.25, 1.92)	1.76 (1.41, 2.19)	115 (4.5)	1.05 (0.85, 1.29)	0.89 (0.72, 1.10)
≥140	114 (0.6)	18 (1.1)	1.96 (1.18, 3.26)	2.11 (1.25, 3.55)	20 (0.8)	1.34 (0.82, 2.19)	1.14 (0.69, 1.89)

Lightweight, small for gestational age (SGA) or low birth weight (LBW); Heavyweight, large for gestational age (LGA) or macrosomia. † The model was adjusted for maternal age, education, parity, gestational age of hemoglobin measurement, neonatal gender, gestational age at delivery and mode of delivery. ‡ The model was additionally adjusted for maternal BMI in the first trimester and weight gain during pregnancy.

Table S6. Associations between the change in maternal hemoglobin concentrations and adverse birth outcomes in pregnant women who delivered their baby after 37 weeks of gestational age.

ΔHb	Normalweight	Lightweight			Heavyweight		
	N (%)	N (%)	Model 1 † OR (95% CI)	Model 2 ‡ OR (95% CI)	N (%)	Model 1 † OR (95% CI)	Model 2 ‡ OR (95% CI)
Hbfs							
Q1 (−50~−17)	4257 (25.2)	379 (24.4)	Ref.	Ref.	704 (30.3)	Ref.	Ref.
Q2 (−17~−11)	4312 (25.6)	366 (23.6)	0.96 (0.82, 1.13)	1.01 (0.87, 1.19)	626 (26.9)	0.91 (0.81, 1.03)	0.84 (0.74, 0.95)
Q3 (−11~−5)	4340 (25.7)	407 (26.2)	1.07 (0.91, 1.25)	1.17 (0.99, 1.38)	521 (22.4)	0.75 (0.66, 0.86)	0.65 (0.56, 0.74)
Q4 (−5~50)	3955 (23.5)	400 (25.8)	1.17 (0.98, 1.40)	1.35 (1.12, 1.62)	476 (20.5)	0.75 (0.64, 0.87)	0.61 (0.52, 0.71)
Hbft							
Q1 (−50~−16)	4830 (26.4)	345 (20.4)	Ref.	Ref.	817 (32.3)	Ref.	Ref.
Q2 (−16~−9)	4500 (24.6)	400 (23.7)	1.34 (1.15, 1.57)	1.38 (1.18, 1.62)	612 (24.2)	0.82 (0.73, 0.92)	0.78 (0.69, 0.88)
Q3 (−9~−2)	4458 (24.3)	431 (25.5)	1.53 (1.30, 1.80)	1.63 (1.38, 1.92)	561 (22.2)	0.77 (0.68, 0.88)	0.71 (0.62, 0.81)
Q4 (−2~50)	4536 (24.8)	513 (30.4)	1.92 (1.60, 2.29)	2.10 (1.75, 2.51)	538 (21.3)	0.74 (0.64, 0.86)	0.64 (0.55, 0.75)
Hbst *							
Q1 (−50~−3.5)	4309 (26.4)	302 (20.2)	Ref.	Ref.	666 (29.7)	Ref.	Ref.
Q2 (−3.5~2)	4393 (26.9)	387 (25.9)	1.23 (1.05, 1.44)	1.23 (1.05, 1.44)	595 (26.6)	0.91 (0.81, 1.03)	0.93 (0.82, 1.05)
Q3 (2~7)	3666 (22.5)	369 (24.7)	1.43 (1.22, 1.68)	1.41 (1.20, 1.66)	445 (19.9)	0.82 (0.72, 0.93)	0.83 (0.72, 0.95)
Q4 (7~50)	3940 (24.2)	438 (29.3)	1.58 (1.35, 1.85)	1.52 (1.30, 1.78)	534 (23.8)	0.95 (0.84, 1.08)	0.99 (0.87, 1.13)

Lightweight, small for gestational age (SGA) or low birth weight (LBW); Heavyweight, large for gestational age (LGA) or macrosomia; Hbfs, Hb in late-second minus Hb in first; Hbft, Hb in late-third minus Hb in late-third; Hbst, Hb in late-second minus Hb in late-third; Q, quartile. † The model was adjusted for maternal age, education, parity, weeks of gestation at hemoglobin measurement, neonatal gender, gestational age at delivery, mode of delivery and Hb concentration in the first trimester. ‡ The model was additionally adjusted for maternal BMI in the first trimester and weight gain during pregnancy. * Model 1 was adjusted for maternal age, education, parity, weeks of gestation at hemoglobin measurement, neonatal gender, gestational age at delivery, mode of delivery and Hb concentration in the late-second trimester.

Table S7. Associations between the maternal hemoglobin concentration in different periods and adverse birth weight outcomes in pregnant women who had at least one record of Hb concentration during each defined period.

Hemoglobin	Normalweight	Lightweight			Heavyweight		
	N (%)	N (%)	Model 1 † OR (95% CI)	Model 2 ‡ OR (95% CI)	N (%)	Model 1 † OR (95% CI)	Model 2 ‡ OR (95% CI)
First trimester							
<100	120 (1.1)	13 (1.2)	1.00 (0.55, 1.81)	0.98 (0.54, 1.78)	13 (0.8)	0.93 (0.51, 1.67)	0.88 (0.48, 1.62)
100~109	657 (5.8)	62 (5.5)	0.91 (0.68, 1.22)	0.91 (0.68, 1.21)	87 (5.4)	1.12 (0.87, 1.45)	1.19 (0.92, 1.54)
110~119	2799 (24.6)	290 (25.7)	Ref.	Ref.	344 (21.5)	Ref.	Ref.
120~129	4640 (40.9)	451 (40.0)	0.94 (0.81, 1.10)	0.98 (0.84, 1.15)	656 (41.1)	1.14 (0.99, 1.32)	1.06 (0.92, 1.23)
130~139	2558 (22.5)	244 (21.7)	0.94 (0.78, 1.12)	1.00 (0.83, 1.20)	391 (24.5)	1.21 (1.03, 1.42)	1.09 (0.93, 1.29)
≥140	581 (5.1)	67 (5.9)	1.15 (0.87, 1.53)	1.25 (0.93, 1.66)	106 (6.6)	1.42 (1.11, 1.80)	1.16 (0.90, 1.50)
Early-second trimester							
<100	279 (2.5)	30 (2.7)	0.95 (0.64, 1.42)	0.88 (0.59, 1.31)	43 (2.7)	1.16 (0.82, 1.63)	1.30 (0.91, 1.84)
100~109	1768 (15.6)	158 (14.0)	0.86 (0.71, 1.05)	0.83 (0.68, 1.00)	229 (14.3)	0.96 (0.81, 1.12)	1.06 (0.90, 1.26)
110~119	4568 (40.2)	468 (41.5)	Ref.	Ref.	634 (39.7)	Ref.	Ref.
120~129	3644 (32.1)	347 (30.8)	0.93 (0.80, 1.08)	0.99 (0.85, 1.14)	535 (33.5)	1.02 (0.90, 1.16)	0.93 (0.82, 1.06)
130~139	920 (8.1)	102 (9.1)	1.10 (0.88, 1.38)	1.22 (0.97, 1.54)	131 (8.2)	0.97 (0.79, 1.19)	0.82 (0.66, 1.01)
≥140	176 (1.5)	22 (2.0)	1.23 (0.78, 1.95)	1.37 (0.86, 2.19)	25 (1.6)	1.06 (0.69, 1.64)	0.86 (0.55, 1.35)
Mediate-second trimester							
<100	474 (4.2)	46 (4.1)	0.96 (0.69, 1.32)	0.84 (0.61, 1.17)	60 (3.8)	0.94 (0.71, 1.25)	1.13 (0.84, 1.52)
100~109	2836 (25.0)	278 (24.7)	1.01 (0.86, 1.17)	0.95 (0.81, 1.11)	412 (25.8)	1.03 (0.90, 1.17)	1.17 (1.02, 1.34)
110~119	5184 (45.7)	504 (44.7)	Ref.	Ref.	745 (46.6)	Ref.	Ref.
120~129	2416 (21.3)	249 (22.1)	1.05 (0.89, 1.23)	1.12 (0.95, 1.32)	320 (20.0)	0.91 (0.79, 1.06)	0.82 (0.71, 0.95)
130~139	370 (3.3)	43 (3.8)	1.24 (0.89, 1.73)	1.32 (0.94, 1.85)	49 (3.1)	0.87 (0.64, 1.19)	0.73 (0.53, 1.01)
≥140	75 (0.7)	7 (0.6)	0.90 (0.41, 1.98)	1.00 (0.45, 2.21)	11 (0.7)	1.05 (0.55, 2.00)	0.86 (0.44, 1.67)
Late-second trimester							
<100	491 (4.3)	50 (4.4)	0.99 (0.73, 1.35)	0.86 (0.63, 1.18)	83 (5.2)	1.29 (1.00, 1.66)	1.56 (1.20, 2.02)
100~109	3301 (29.1)	299 (26.5)	0.91 (0.78, 1.06)	0.84 (0.72, 0.98)	476 (29.8)	1.06 (0.93, 1.20)	1.21 (1.06, 1.38)
110~119	5265 (46.4)	518 (46.0)	Ref.	Ref.	738 (46.2)	Ref.	Ref.
120~129	2005 (17.7)	220 (19.5)	1.13 (0.95, 1.33)	1.21 (1.02, 1.43)	249 (15.6)	0.86 (0.74, 1.01)	0.78 (0.66, 0.92)
130~139	234 (2.1)	34 (3.0)	1.43 (0.98, 2.09)	1.52 (1.03, 2.24)	45 (2.8)	1.40 (1.00, 1.96)	1.14 (0.80, 1.62)
≥140	59 (0.5)	6 (0.5)	0.98 (0.41, 2.29)	1.07 (0.45, 2.57)	6 (0.4)	0.78 (0.33, 1.84)	0.71 (0.30, 1.69)

Early-third trimester								
<100	457 (4.0)	31 (2.8)	0.65 (0.44, 0.94)	0.59 (0.40, 0.86)	76 (4.8)	1.31 (1.00, 1.70)	1.49 (1.13, 1.95)
100~109	3276 (28.9)	264 (23.4)	0.79 (0.67, 0.92)	0.74 (0.63, 0.87)	519 (32.5)	1.21 (1.07, 1.37)	1.32 (1.16, 1.50)
110~119	5257 (46.3)	520 (46.1)	Ref.	Ref.	700 (43.8)	Ref.	Ref.
120~129	2008 (17.7)	269 (23.9)	1.31 (1.12, 1.54)	1.39 (1.19, 1.64)	256 (16.0)	0.96 (0.82, 1.12)	0.84 (0.72, 0.99)
130~139	295 (2.6)	35 (3.1)	1.20 (0.83, 1.73)	1.25 (0.86, 1.81)	32 (2.0)	0.79 (0.54, 1.16)	0.72 (0.49, 1.06)
≥140	62 (0.5)	8 (0.7)	1.26 (0.60, 2.66)	1.30 (0.61, 2.76)	14 (0.9)	1.81 (0.99, 3.28)	1.57 (0.84, 2.96)
Late-third trimester							
<100	297 (2.6)	28 (2.5)	0.98 (0.65, 1.46)	0.93 (0.62, 1.39)	50 (3.1)	1.27 (0.93, 1.75)	1.38 (1.00, 1.92)
100~109	2687 (23.7)	202 (17.9)	0.80 (0.67, 0.94)	0.76 (0.64, 0.91)	447 (28.0)	1.26 (1.11, 1.43)	1.32 (1.15, 1.51)
110~119	5344 (47.1)	501 (44.5)	Ref.	Ref.	710 (44.5)	Ref.	Ref.
120~129	2492 (21.9)	298 (26.4)	1.26 (1.08, 1.46)	1.31 (1.12, 1.53)	318 (19.9)	0.95 (0.83, 1.10)	0.86 (0.74, 1.00)
130~139	472 (4.2)	84 (7.5)	1.78 (1.38, 2.30)	1.96 (1.51, 2.54)	64 (4.0)	1.01 (0.77, 1.34)	0.86 (0.65, 1.15)
≥140	63 (0.6)	14 (1.2)	2.14 (1.16, 3.95)	2.27 (1.21, 4.25)	8 (0.5)	0.96 (0.46, 2.04)	0.84 (0.39, 1.79)

Lightweight, small for gestational age (SGA) or low birth weight (LBW); Heavyweight, large for gestational age (LGA) or macrosomia. † The model was adjusted for maternal age, education, parity, gestational age of hemoglobin measurement, neonatal gender, gestational age at delivery and mode of delivery. ‡ The model was additionally adjusted for maternal BMI in the first trimester and weight gain during pregnancy.

Table S8. Associations between the change in maternal hemoglobin concentration and adverse birth weight outcomes in pregnant women who had at least one record of Hb concentration during each defined period.

ΔHb	Normalweight	Lightweight			Heavyweight		
	N (%)	N (%)	Model 1 † OR (95% CI)	Model 2 ‡ OR (95% CI)	N (%)	Model 1 † OR (95% CI)	Model 2 ‡ OR (95% CI)
Hbfs							
Q1 (−50~−17)	2926 (25.8)	276 (24.6)	Ref.	Ref.	488 (30.6)	Ref.	Ref.
Q2 (−17~−11)	2941 (26.0)	253 (22.5)	0.93 (0.77, 1.12)	0.98 (0.81, 1.18)	435 (27.3)	0.92 (0.79, 1.06)	0.85 (0.73, 0.99)
Q3 (−11~−5)	2909 (25.7)	312 (27.8)	1.20 (0.99, 1.45)	1.31 (1.08, 1.59)	350 (22.0)	0.74 (0.63, 0.87)	0.65 (0.55, 0.77)
Q4 (−5~50)	2556 (22.6)	282 (25.1)	1.27 (1.02, 1.57)	1.46 (1.18, 1.82)	321 (20.1)	0.77 (0.64, 0.93)	0.62 (0.51, 0.75)
Hbft							
Q1 (−50~−16)	2990 (26.4)	228 (20.3)	Ref.	Ref.	527 (33.1)	Ref.	Ref.
Q2 (−16~−9)	2824 (24.9)	264 (23.5)	1.35 (1.12, 1.64)	1.39 (1.14, 1.69)	379 (23.8)	0.77 (0.66, 0.90)	0.74 (0.63, 0.86)
Q3 (−9~−2)	2768 (24.4)	285 (25.3)	1.59 (1.30, 1.94)	1.67 (1.36, 2.04)	355 (22.3)	0.74 (0.63, 0.87)	0.69 (0.58, 0.81)
Q4 (−2~50)	2757 (24.3)	348 (30.9)	2.08 (1.67, 2.59)	2.24 (1.80, 2.80)	332 (20.8)	0.71 (0.59, 0.86)	0.62 (0.51, 0.75)
Hbst *							
Q1 (−50~−3.5)	2894 (25.5)	224 (19.9)	Ref.	Ref.	459 (28.8)	Ref.	Ref.
Q2 (−3.5~2)	3043 (26.8)	290 (25.8)	1.20 (1.00, 1.44)	1.20 (1.00, 1.44)	434 (27.2)	0.94 (0.82, 1.09)	0.97 (0.83, 1.12)
Q3 (2~7)	2621 (23.1)	261 (23.2)	1.26 (1.04, 1.52)	1.25 (1.03, 1.51)	324 (20.3)	0.82 (0.71, 0.96)	0.85 (0.72, 1.00)
Q4 (7~50)	2784 (24.5)	350 (31.1)	1.57 (1.31, 1.87)	1.51 (1.26, 1.81)	377 (23.7)	0.92 (0.79, 1.07)	0.97 (0.83, 1.13)

Lightweight, small for gestational age (SGA) or low birth weight (LBW); Heavyweight, large for gestational age (LGA) or macrosomia; Hbfs, Hb in late-second minus Hb in first; Hbft, Hb in late-third minus Hb in late-third; Hbst, Hb in late-second minus Hb in late-third; Q, quartile. † The model was adjusted for maternal age, education, parity, weeks of gestation at hemoglobin measurement, neonatal gender, gestational age at delivery, mode of delivery and Hb concentration in the first trimester. ‡ The model was additionally adjusted for maternal BMI in the first trimester and weight gain during pregnancy. * Model 1 was adjusted for maternal age, education, parity, weeks of gestation at hemoglobin measurement, neonatal gender, gestational age at delivery, mode of delivery and Hb concentration in the late-second trimester.
